# P-1490. Health Outcomes and Economic Burden in Commercially- and Medicaid-Insured Infants Diagnosed with Invasive Meningococcal Disease in the United States

**DOI:** 10.1093/ofid/ofaf695.1674

**Published:** 2026-01-11

**Authors:** Oscar Herrera-Restrepo, Elizabeth Packnett, Megan Richards, Elise Kuylen, Tosin Olaiya, Thatiana Pinto, Lindsay Landgrave, Andrew G Allmon

**Affiliations:** GSK, Philadelphia, PA; Merative, Ann Arbor, Michigan; Merative, Ann Arbor, Michigan; GSK, Philadelphia, PA; GSK, Philadelphia, PA; GSK, Philadelphia, PA; GSK, Philadelphia, PA; GSK, Philadelphia, PA

## Abstract

**Background:**

While uncommon, invasive meningococcal disease (IMD) is severe and progresses rapidly. In the United States (US), the highest IMD incidence rates are reported among infants. In this study, we describe health outcomes and economic burden among US infants (< 1 year) diagnosed with IMD.

**Figure:**
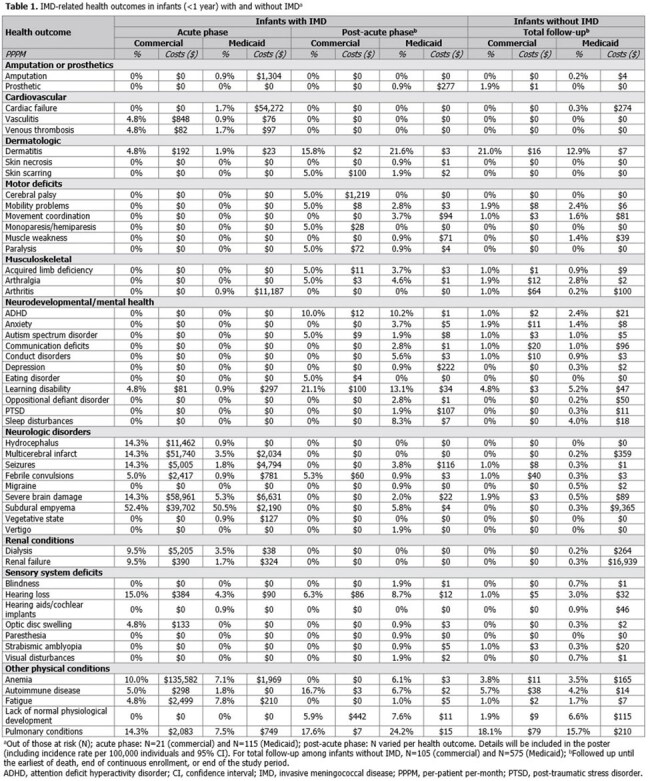

**Methods:**

In this retrospective study, claims data of commercially- and Medicaid-insured infants from 01/2005–12/2022 were analyzed. Infants with a primary IMD diagnosis were identified using inpatient admissions data and matched 1:5 with infants without IMD. Health outcomes were tracked, and for infants with IMD were stratified by acute phase (index date to 30 days post-discharge) and post-acute phase (from acute phase to death, insurance disenrollment, or end of study period). A variable follow-up period from index was used to assess IMD-related direct medical and out-of-pocket costs, reported per-patient per-month (PPPM).

**Results:**

In the commercial (N=7.2 million) and Medicaid (N=7.4 million) cohorts, 21 and 115 infants were diagnosed with IMD, respectively. Median follow-up length across cohorts and IMD status was 426–779 days. Infants with IMD (in both acute and post-acute phases) had a higher occurrence (≥0.5%) of many negative health outcomes versus (vs.) matched infants (Table 1). IMD-related inpatient costs (mean [standard deviation]) were significantly higher in infants with IMD than matched infants (commercial: $17,095 [$41,590] vs. $7 [$48]; Medicaid: $3,750 [$15,262] vs. $22 [$228]; both p< 0.001). IMD-related office visit, specialist visit, and surgical procedure costs were significantly higher for commercially-insured infants with IMD than matched infants ($85 [$129] vs. $39 [$37]; $33 [$67] vs. $9 [$24]; $860 [$2,879] vs. $0 [$0], respectively; all p≤ 0.006), as were out-of-pocket inpatient, outpatient, and surgical procedure costs (not significant in Medicaid cohort). Other IMD-related outpatient service costs were significantly higher among infants with IMD than matched infants across cohorts.

**Conclusion:**

Individuals who had IMD during infancy experienced substantial negative health outcomes and economic burden. Preventing IMD in early childhood may help to reduce negative impacts on individuals, families, and healthcare systems.

Funding: GSK VEO-000995

**Disclosures:**

Oscar Herrera-Restrepo, PhD, GSK: Employee|GSK: Stocks/Bonds (Public Company) Elizabeth Packnett, MPH, GSK: Grant/Research Support|Merative: Employee Megan Richards, PhD, MPH, GSK: Grant/Research Support|Merative: Employee Elise Kuylen, PhD, GSK: Employee|GSK: Stocks/Bonds (Public Company) Tosin Olaiya, MBChB, MSc, GSK: Employee|GSK: Stocks/Bonds (Public Company) Thatiana Pinto, PhD, GSK: employee|GSK: Stocks/Bonds (Public Company) Lindsay Landgrave, PharmD, GSK: Employee|GSK: Stocks/Bonds (Public Company) Andrew G. Allmon, DrPH, GSK: Employee|GSK: Stocks/Bonds (Public Company)

